# Caveolin-1, cellular senescence and pulmonary emphysema

**DOI:** 10.18632/aging.100079

**Published:** 2009-08-10

**Authors:** Daniela Volonte, Ferruccio Galbiati

**Affiliations:** Department of Pharmacology & Chemical Biology, University of Pittsburgh School of Medicine, Pittsburgh, PA 15261, USA

**Keywords:** Caveolin, oxidative stress, premature senescence, p53

## Abstract

Caveolae are vesicular invaginations of the plasma membrane. Caveolin-1 is the structural protein component of caveolae. Caveolin-1 participates in signal transduction processes by acting as a scaffolding protein that concentrates, organizes and functional regulates signaling molecules within caveolar membranes. Cigarette smoke, a source of oxidants, is an environmental hazard that causes pulmonary emphysema. Recently, we reported that the development of cigarette smoking-induced pulmonary emphysema was inhibited in caveolin-1 null mice, which do not express caveolin-1. We demonstrated that lack of caveolin-1 expression in lung fibroblasts dramatically inhibited premature senescence induced by oxidants contained in cigarette smoke. Mechanistically, we uncovered that premature senescence of lung fibroblasts induced by oxidative stress occurred through activation of an ataxia telangiectasia-mutated (ATM)/p53-depedent pathway following sequestration of the catalytic subunit of protein phosphatase 2A (PP2A-C), an inhibitor of ATM, by caveolin-1 into caveolar membranes. We propose caveolin-1 as a key player of a novel signaling pathway that links cigarette smoke to premature senescence of lung fibroblasts and development of pulmonary emphysema.

## Caveolae and caveolin-1

Caveolae, 50-100 nm flask-shaped invaginations of the plasma membrane, are found in many cell types, including fibroblasts [1]. Caveolae represent a subgroup of lipid rafts, which are microdomains of the plasma membrane enriched in cholesterol, sphingolipids and glycosyl phosphatidylinositol anchored proteins [2]. The presence of the structural protein caveolin-1 drives the formation of the plasma membrane invaginations and makes caveolae unique among lipid rafts. Caveolae have been implicated in numerous cellular functions, including signal transduction, cellular metabolism, vesicle trafficking, cholesterol homeostasis, endothelial transcytosis, and tumor suppression [2-4]. Caveolin-1 acts as a scaffolding protein to compartmentalize and functionally regulate signaling molecules within caveolar membranes [2].

## Caveolin-1 regulates stress-induced premature senescence (SIPS)

Several theories have been proposed in the past to explain why and how living organisms can not escape aging. The "free radical theory" of aging was proposed by Denham Harman in the fifties and is based on the concept that normal aging occurs as the result of tissue damages inflicted by reactive oxygen species (ROS) [5]. In support of this theory, increased oxidative damage of DNA, proteins, and lipids have been reported in aged animals [6]. Thus, endogenous and exogenous stimuli may significantly increase oxidant levels within the cell and induce a series of cellular damages.

Most cells cannot divide indefinitely due to a process termed cellular senescence [7-13]. Growth arrest is associated with well-defined biochemical alterations. These include cell cycle arrest, increased p53 activity, increased p21^Waf1/Cip1^ and p16 protein expression, and hypo-phosphorylation of pRb [7-11]. Interestingly, subcytotoxic oxidative stress has been shown to accelerate the induction of cellular senescence in a number of cell types in culture, including fibroblasts [14-16]. Thus, investigating the signaling machinery that regulates the ability of free radicals to induce premature senescence in cell culture models will contribute to a better understanding of the more complicated aging process.

Our group has demonstrated a key role of caveolin-1 in the induction of cellular senescence. We showed that over-expression of caveolin-1 in mouse embryonic fibroblasts was sufficient to induce premature senescence, as demonstrated by cell cycle arrest in the G_0_/G_1_ phase of the cell cycle, a reduced proliferative lifespan, up-regulation of p21^Waf1/Cip1^, development of senescence-like cell morphology and senescence-associated increase in β-galactosidase activity [1,17]. We also showed that caveolin-1 plays a direct role in oxidative stress-induced premature senescence, as demonstrated by inhibition of SIPS in mouse embryonic fibroblasts derived from caveolin-1 null mice, which do not express caveolin-1, and NIH 3T3 cells harboring antisense caveolin-1 [1,18].

Since over-expression of caveolin-1 was sufficient to induce premature senescence and caveolin-1 expression was required for SIPS, we also asked whether free radicals had an effect on endogenous caveolin-1 expression. We found that sub-cytotoxic oxidative stress up-regulated caveolin-1 protein expression through activation of the caveolin-1 gene promoter in a p38 mitogen-activated protein kinase/Sp1-dependent manner [19].

What is the molecular mechanism underlying caveolin-1-mediated SIPS? We found that caveolin-1 is a novel binding protein for Mdm2, a negative regulator of p53. We showed that after oxidative stress caveolin-1 sequestered Mdm2 away from p53, leading to stabilization of p53 and up-regulation of p21^Waf1/Cip1^. Consistent with these data, expression of a peptide corresponding to the Mdm2 binding domain of caveolin-1 was sufficient to up-regulate p53 and p21^Waf1/Cip1^ protein expression and induce premature senescence. Thus, we propose caveolin-1 as a signaling molecule whose ability to activate the p53 pathway is critical for stress-induced premature senescence.

Our results have been supported by studies showing that senescent human diploid fibroblasts express higher levels of caveolin-1, as compared to younger human diploid fibroblasts [20]. Up-regulation of caveolin-1 was associated to a significant inhibition of EGF-stimulated ERK-1/2 phosphorylation [20]. Caveolin-1 has also been shown to play an important role in senescence-associated morphological changes by regulating focal adhesion kinase activity and actin stress fiber formation in senescent cells [21]. In addition, it has been shown that replicative senescent cells re-enter the cell cycle upon EGF stimulation after down-regulation of caveolin-1 [22]. Together, these data indicate that caveolin-1 plays a key role in the signal transduction events leading to cellular senescence.

## Role of caveolin-1 in cigarette smoking-induced pulmonary emphysema

Pulmonary emphysema is an age-related disease of the lungs. It occurs after a prolonged period of cigarette smoking. Pulmonary emphysema is characterized by alveolar destruction, airspace enlargement and reduction of alveolar capillary exchange area. Because cigarette smoke is enriched in potent oxidants, oxidative stress is believed to play a key role in the pathogenesis of emphysema [23,24]. The classical concept of the pathogenesis of emphysema was based on lung inflammation caused by cigarette smoke and environmental pollutants, leading to a protease/antiprotease imbalance [25]. However, cigarette smoke has been shown to promote premature senescence of lung fibroblasts in culture [26]. In addition, a reduced proliferation rate [27,28], lower number of population doubling in culture [27], and increased senescence-associated β-galactosidase activity [29] were observed in lung fibroblasts from patients with emphysema. Since fibroblasts play a structural role that is necessary for proper lung integrity, the presence of senescent fibroblasts may affect tissue microbalance and structural maintenance of the lungs. In addition, senescent cells can secrete matrix metalloproteases [30] and inflam-matory cytokines [31,32] that could enhance the protease/antiprotease imbalance and fuel the abnormal inflammatory response in the lungs, respectively. Thus, accumulation of senescent fibroblasts may contribute to the development of pulmonary emphysema. However, the molecular mechanisms linking cigarette smoke to premature senescence of lung cells and emphysema remain to be fully identified.

We have recently shown that cigarette smoke extracts induced premature senescence of lung fibroblasts in a caveolin-1-dependent manner [33]. More specifically, the number of senescent cells was dramatically reduced and the up-regulation of p53 and p21^Waf1/Cip1^ was significantly inhibited in lung fibroblasts derived from caveolin-1 null mice, which do not express caveolin-1, after treatment with cigarette smoke extracts. Co-treatment with antioxidants prevented the ability of cigarette smoke extracts to induce premature senescence of lung fibroblasts, suggesting that oxidants contained in cigarette smoke extracts were responsible for the observed senescent phenotype. We also identified a mechanism through which oxidative stress induces premature senescence of lung fibroblasts. Free radicals have been shown to activate the ATM protein kinase [34], a key activator of p53. We found that sequestration of PP2A-C, an ATM inhibitor, into caveolar membranes was required for the activation of ATM and up-regulation of p53 in wild type fibroblasts upon oxidative stress [33]. Cigarette smoke extracts failed to activate ATM and up-regulate p53 in caveolin-1 null lung fibroblasts [33].

Does a lack of caveolin-1 prevent cigarette smoke-induced activation of p53 and premature senescence *in vivo*? When caveolin-1 null mice were exposed to cigarette smoking for either 6 weeks or 6 months, premature senescence of lung fibroblasts and activation of the p53 pathway were significantly prevented, as compared to wild type mice [33]. Because exposure to cigarette smoking for 6 months has been shown to induce pulmonary emphysema in mice, we examined the lung phenotype of caveolin-1 null mice exposed to cigarette smoking for 6 months. We found that, in contrast to wild type mice, the development of pulmonary emphysema was significantly inhibited in caveolin-1 null mice [33]. Senescent fibroblasts were observed in the lungs of wild type mice after only 6 weeks of exposure to cigarette smoking while pulmonary emphysema was morphologically detectable after 6 months of exposure. Considering that a lack of caveolin-1 prevented both premature senescence of lung fibroblasts and development of pulmonary emphysema, we propose a model in which oxidants contained in cigarette smoke induce premature senescence of lung fibroblasts in a caveolin-1/ATM/p53-dependent manner and that senescent lung fibroblasts contribute to the pathogenesis of pulmonary emphysema.

**Figure 1.**
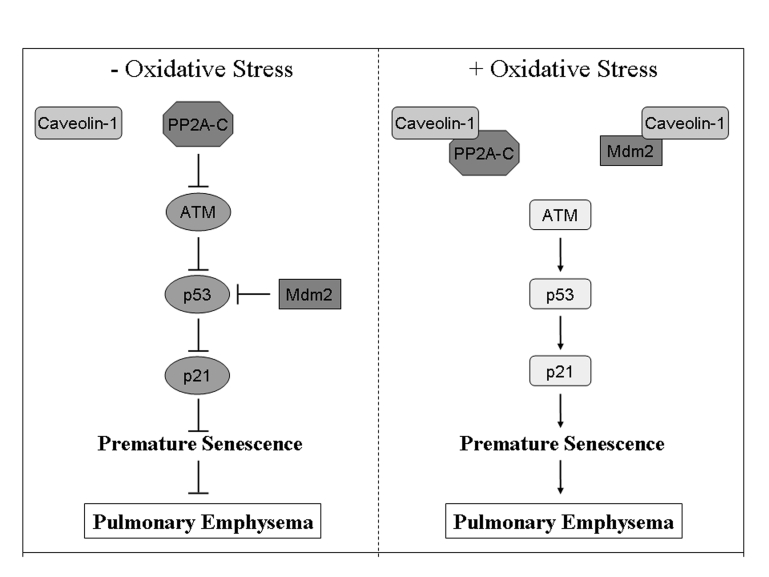
Schematic diagram summarizing the caveolin-1-dependent activation of the p53/p21 ^Waf1/Cip1^/senescence pathway after oxidative stress. In resting cells, PP2A-C-dependent inhibition of ATM prevents the activation of p53. In addition, p53 is directly inhibited by binding to Mdm2. Oxidative stress promotes the sequestration of PP2A-C and Mdm2 by caveolin-1 leading to activation of p53 and its downstream target p21^Waf1/Cip1^, and induction of premature senescence. Activation of the p53/p21^Waf1/Cip1^/senescence pathway after oxidative stress is inhibited in cells lacking caveolin-1 expression. We suggest that activation of this pathway in lung fibroblasts by oxidants contained in cigarette smoke contributes to the development of pulmonary emphysema. Adapted from [33].

Activation of ATM following the caveolin-1-mediated sequestration of PP2A-C may not be the only mechanism employed by cigarette smoke to activate p53. As mentioned earlier, we have shown that caveolin-1 activated the p53 pathway after oxidative stress (hydrogen peroxide was used a source of free radicals in these experiments) through an Mdm2-dependent pathway [18]. Although we have not proved it directly, we speculate that oxidants contained in cigarette smoke may activate p53 through both caveolin-1/ATM- and caveolin-1/Mdm2-dependent mechanisms.

Lungs from caveolin-1 null mice have marked hypercellularity resulting in thickening of the alveolar wall and constriction of alveolar spaces [35]. This hypercellularity can be correlated with the excessive proliferation of MEFs derived from caveolin-1 null mice that is observed in cell culture models and is consistent with data proposing caveolin-1 as a tumor suppressor [36]. The hyperproliferation of lung cells observed in caveolin-1 null mice may counterbalance alveolar destruction and airspace enlargement induced by cigarette smoking, contributing to explain the lower number of senescent cells and the milder emphysematous phenotype that we have observed in these mice.

## Conclusive remarks

Based on our findings, we propose caveolin-1 as a novel upstream positive regulator of p53 in the signaling pathway that leads to premature senescence of lung fibroblasts upon oxidant stimulation and, eventually, to pulmonary emphysema. We currently do not know whether cigarette smoke induces premature senescence of other lung cell types, such as alveolar epithelial cells, which mediate oxygen absorption. Since caveolin-1 is also endogenously expressed in these cells, it is possible that caveolin-1 may also mediate oxidative stress-induced premature senescence of lung epithelial cells *in vivo* and that senescent alveolar epithelial cells may contribute to the development of pulmonary emphysema. Thus, one may envision a therapeutic intervention aimed at lowering caveolin-1 expression in lung cells for the treatment and/or prevention of the tissue damages that are caused by cigarette smoking. However, given the role that caveolin-1 plays as a tumor suppressor in certain forms of cancer, such as breast cancer, and that most types of cancer are of epithelial origin, we can not rule out the possibility that the indiscriminate down-regulation of caveolin-1 expression in lung cells may limit emphysema but promote the development of lung cancer. Therefore, a targeted down-regulation of caveolin-1 expression in lung fibroblasts may be a strategic approach to limit emphysema without promoting tumor development.
